# The Potential of Shortening the Adaptation of Nellore Cattle to High-Concentrate Diets Using Only Virginiamycin as Sole Feed Additive

**DOI:** 10.3389/fvets.2021.692705

**Published:** 2021-08-02

**Authors:** André L. N. Rigueiro, Mariana M. Squizatti, Antonio M. Silvestre, Ana C. J. Pinto, Daniela D. Estevam, Luana D. Felizari, Evandro F. F. Dias, Breno L. Demartini, Ana B. P. C. Nunes, Victor C. M. Costa, Eduardo L. Caixeta, Pedro F. Santi, Carlos H. G. Soares, Mario D. B. Arrigoni, Danilo D. Millen

**Affiliations:** ^1^Department of Breeding and Animal Nutrition, School of Veterinary Medicine and Animal Science, São Paulo State University (UNESP), Botucatu, Brazil; ^2^Department of Animal Production, College of Agricultural and Technological Sciences, São Paulo State University (UNESP), Dracena, Brazil

**Keywords:** antibiotic, *Bos indicus*, epithelium, fat, ionophore

## Abstract

Feedlot cattle are usually adapted to high-concentrate diets containing sodium monensin (MON) in more than 14 days. However, for finishing diets with lower energy content, the use of MON during adaptation may hold dry matter intake (DMI), and virginiamycin (VM) may be an alternative. This study was designed to determine the potential of shortening the adaptation of Nellore cattle to high-concentrate diets using only VM as a sole feed additive relative to feedlot performance, feeding behavior, and ruminal and cecum morphometrics. The experiment was designed as a completely randomized block replicated six times (four animals/pen) in which 120 Nellore bulls (390.4 ± 19.0 kg) were fed in 30 pens for 111 days according to the following treatments: (1) MON and adaptation for 14 days (MON14), (2) MON + VM and adaptation for 14 days (MONVM14), (3) VM and adaptation for 14 days (VM14), (4) VM and adaptation for 9 days (VM9), and (5) VM and adaptation for 6 days (VM6). At the end of the adaptation, 30 animals (*n* = 1 per pen) were randomly slaughtered for rumen and cecum evaluations. The remaining 90 bulls were harvested at the end of the study. No effects of treatments were observed (*P* < 0.10) for final body weight, average daily gain (ADG), and hot carcass weight (HCW). Cattle fed VM14 presented a greater (*P* ≤ 0.03) DMI, expressed as percent of body weight (BW), than animals fed either MON14 or MONVM14; however, cattle fed either MON14 or MONVM14 improved (*P* ≤ 0.02) the gain-to-feed ratio (G/F) by 10.4 or 8.1%, respectively, when compared to bulls fed VM14. Bulls fed VM14 had smaller (*P* < 0.05) papillae area (0.34 vs. 0.42 cm^2^) and rumen absorptive surface area (28.9 vs. 33.8 cm^2^) than those fed MON14. The shortening of the adaptation period linearly decreased the 12th rib fat (*P* = 0.02) and biceps femoris fat daily gain (*P* = 0.02) of Nellore bulls fed only VM, which linearly decreased the final biceps femoris fat thickness (*P* < 0.01). Feedlot cattle fed VM as a sole feed additive should not be adapted to high-concentrate diets in less than 14 days. Regardless of either adaptation length or feed additive, feedlot cattle need at least 14 days to adapt to finishing diets.

## Introduction

Adaptation studies have been conducted previously in Brazil to determine the most appropriate adaptation length to high-concentrate diets for Nellore cattle (1–5). Based on the results of these studies, Nellore cattle should not be adapted in less than 14 days, regardless of the type of adaptation protocol adopted, corn processing method, and nutritional background. Other authors (6) also reported that feedlot cattle in North America could not be adapted in less than 14 days without impairing the overall performance and health.

However, all adaptation studies conducted previously in Brazil with Nellore cattle used feedlot diets containing sodium monensin (MON) as the sole feed additive. Monensin is known for its negative effect on dry matter intake (DMI), which typically results in a positive effect on the feed efficiency of feedlot cattle (7). Furthermore, the DMI is usually lower during adaptation when compared to the finishing period (8), and the use of a feed additive, such as the MON, may decrease even further the DMI in this period. Since finishing diets in Brazil contain less energy than a typical feedlot diet in North America (9, 10), the use of a feed additive that does not impact the DMI, especially during adaptation, may be recommended.

Among potential feed additives that might be recommended for feedlot cattle, virginiamycin (VM) is a growth promoter that does not decrease the DMI and improves feedlot performance (11), and it was the second feed additive mostly used by Brazilian nutritionists in 2019 (12). When VM was evaluated as an alternative to MON (8), authors reported that Nellore bulls fed VM as the sole feed additive during adaptation reached a DMI of 2% of the initial body weight (BW) in 4.3 days on average, whereas those fed MON needed 20.7 days to reach a similar intake. The DMI is an important indicator to evaluate how well cattle are either accepting or adapted to the diets (6), and the faster cattle reach a DMI of 2% of BW, the more adapted they are to the diets. Although VM and MON show similar effects on ruminal fermentation patterns, the effect of VM on the DMI of feedlot cattle is minimum even at high doses [19–27 mg/kg (11)]. Therefore, the use of VM as the sole feed additive in finishing diets containing a moderate amount of energy might change the current recommendation of at least 14 days to adapt feedlot Nellore cattle to high-concentrate diets.

Thus, we hypothesized that the adaptation period could be shortened to 9 days or even 6 days when VM is used in finishing diets as the sole feed additive. This study was designed to determine the effects of shortening the adaptation length on feedlot performance, carcass traits, feeding behavior, and ruminal and cecum morphometrics of Nellore cattle fed high-concentrate diets containing only VM as a feed additive.

## Materials and Methods

The protocols and procedures followed in this study were approved by the São Paulo State University Ethical Committee for Animal Research (protocol CEUA 154/2016). The study was conducted at the São Paulo State University feedlot, Dracena Campus, Brazil.

### Animals and Treatments

The study was conducted using 120 yearling Nellore bulls (initial BW = 390.41 ± 19.03 kg) that were housed in 30 pens (*n* = 4 per pen; 1.5 m of linear bunk space and 18 m^2^ of pen space per animal) according to the following treatments: (1) MON [27 mg/kg of dry matter (DM)] and 14-day adaptation (MON14), (2) MON (27 mg/kg of DM) + VM (25 mg/kg of DM) and 14-day adaptation (MONVM14), (3) VM (25 mg/kg of DM) and 14-day adaptation (VM14), (4) VM (25 mg/kg of DM) and 9-day adaptation (VM9), and (5) VM (25 mg/kg of DM) and 6-day adaptation (VM6).

### Feeding and Management Description

At the beginning of the study, all yearling bulls were dewormed and vaccinated (tetanus, bovine viral diarrhea virus, seven-way *Clostridium* sp.; Cattlemaster and Bovishield, Pfizer Animal Health, New York, NY). The yearling bulls were fed *ad libitum* three times per day at 08:00 h (35% of total ration), 11:00 h (20% of total ration), and 16:00 h (45% of total ration) with free-choice access to a water trough (3.00 × 0.80 × 0.20 m).

The experimental diets were composed of sugarcane bagasse, *Cynodon dactylon* hay, finely ground corn grain, soybean meal, mineral supplement, and urea. The experimental diets were formulated according to Fox et al. (13) using the Large Ruminant Nutrition System and are shown in Table 1. The step-up adaptation program consisted of *ad libitum* intake with increasing levels of concentrate ingredients until reaching the concentrate level for the finishing diet (84%). Therefore, the adaptation protocol was conducted as follows: cattle adapted for 6 days were fed diets containing 66, 72, and 78% concentrate for 2 days each and animals adapted for 9 days received diets containing 66, 72, and 78% concentrate for 3 days each, whereas cattle adapted for 14 days were fed 66% concentrate for 5 days, 72% concentrate for 4 days, and 78% concentrate for 5 days.

**Table 1:** Feed ingredients and chemical composition of high-concentrate diets fed to Nellore yearling bulls during adaptation and finishing periods.

**Item**	**Percent of concentrate**			
	**66**	**72**	**78**	**84**
**Ingredients, % of DM**				
Sugarcane bagasse	20.00	18.00	16.00	12.00
*Cynodon dactylon* hay	15.00	10.00	5.00	2.00
Finely ground corn grain	41.80	50.00	59.60	70.00
Soybean meal	20.00	18.70	16.00	12.55
Supplement^a^	2.50	2.50	2.50	2.50
Urea	0.70	0.80	0.90	0.95
**Nutrient content, % of DM** ^**b**^				
Dry matter	46.00	48.00	51.00	57.00
Total digestible nutrients	64.00	67.00	70.00	74.00
Crude protein	15.60	15.60	15.20	14.60
Neutral detergent fiber	41.40	36.60	31.40	14.60
Non-fiber carbohydrates	38.00	43.00	49.00	55.00
peNDF^c^	28.00	23.00	18.00	13.00
Neg, Mcal/kg	1.00	1.08	1.15	1.26
Ca	0.60	0.58	0.56	0.54
P	0.40	0.41	0.42	0.42

*DM, dry matter; Neg, net energy for gain (Mcal)*.

a *The supplement contents were as follows: Ca, 182g/kg of DM; P, 40.5g/kg of DM; Mg, 7.7g/kg of DM; K, 0.5g/kg of DM; Na, 82.2g/kg of DM; Cl, 126.5g/kg of DM; S, 16g/kg of DM; Co, 27.50 mg/kg of DM; Cu, 754.17 mg/kg of DM; Fe, 2498 mg/kg of DM; I, 37.29 mg/kg of DM; Mn, 740 mg/kg of DM; Se, 6.20 mg/kg of DM; Zn, 1,790 mg/kg of DM. Monensin (Bovensin 200; Phibro Animal Health Corporation, Guarulhos, São Paulo, Brazil) was added at 1,000 mg/kg of supplement and Virginiamycin (V-Max 2; Phibro Animal Health Corporation, Guarulhos, São Paulo, Brazil) was added at 833 mg/kg of supplement and offered to yearling bulls according to the treatments*.

b *Estimated by equations according to Large Ruminant Nutrition System (13)*.

c *Physically effective NDF determined according to method described by Heinrichs and Kononoff (14)*.

### Feedlot Performance and Carcass Traits

At the beginning (day 0) and at the end of the experimental period (day 111), the bulls were withheld from feeding for 16 h before BW assessment. Similarly, all animals were weighed on days 28, 56, and 84 of the study, but in these assessments, they were not withheld from feeding, and then 4% of the BW of the animals was deducted and partial gains were calculated within each period (every 28 days). The DMI that was expressed both in kilograms and as a percentage of BW was calculated daily by weighing the ration offered and orts before the next morning delivery. Consequently, the average daily gain (ADG) and the gain-to-feed ratio (G/F) were calculated at the end of the experimental period, as well as the cost to gain a kilogram of BW (R$ per kilogram of DM^*^DMI/ADG) expressed in Brazilian Reais. The DMI variation was calculated for each period as the difference in intake between consecutive days throughout the study (15). Daily DM intake variation was expressed in kilograms and as a percentage of variation. In order to estimate the net energy for maintenance (NEm) and the net energy for gain (NEg) of the experimental diets, the methods described by Lofgreen and Garret (16), NRC (17), and Zinn and Shen (18) were used.

The 12th rib fat thickness, the biceps femoris (BF) fat thickness, the longissimus muscle (LM) area, and the marbling were measured *via* ultrasound at the beginning (day 0) and at the end of the study (day 111) following the method described by Perkins et al. (19) using BIA software (Designer Genes Technologies, Harrison, AR, USA). The images were collected using an Aloka SSD-1100 Flexus RTU unit (Aloka Co. Ltd., Tokyo, Japan) with a 17.2-cm, 3.5-MHz probe.

At the end of the adaptation period (6, 9, or 14 days), 30 animals (*n* = 1 per pen) were randomly chosen to be slaughtered for liver abscess, rumen, and cecum morphometric evaluations. The remaining 90 Nellore yearling bulls (*n* = 3 per pen) were harvested at the end of the study. The final BW was obtained at the feedlot prior to truck loading, and the cattle were then transported for 350 km (about 5 h) to a commercial abattoir. At the abattoir, a captive bolt device was used for stunning the cattle prior to slaughter. The hot carcass weight (HCW) was obtained after kidney, pelvic, and heart fat removal. The dressing percentage was calculated by dividing the HCW by the final BW.

### Feeding Behavior and Particle Sorting

The cattle were submitted to two visual observations to evaluate the feeding behavior and particle sorting. The days of evaluation consisted of observations every 5 min during a period of 24 h. The visual observations were performed on the next day after the end of adaptation (7, 10, or 15 days) and on day 60 of the study according to Pereira et al. (5). During the observations, time spent eating, ruminating, and resting (expressed in minutes) and the number of meals per day were recorded. A meal was considered the non-interrupted time that an animal stayed at the feed bunk eating the ration. The final data for each variable just described represented the average of animals measured in each pen. Similarly, in each 24-h observation period, samples of diets and orts were collected for future analyses of DM (20) and neutral detergent fiber (NDF), assayed with a heat-stable amylase and expressed exclusive of residual ash [aNDFom-NDF (21)] in order to determine DMI and NDF intake on the day of the feeding behavior evaluations.

The meal length in minutes was calculated by dividing the total time spent eating in a day by the number of meals per day. The DMI per meal in kilograms was calculated by dividing the DMI by the number of meals per day. In addition, time spent eating and time spent ruminating data were used to calculate the eating rate of DM (ERDM; time spent eating/DMI) and the rumination rate of DM (RRDM; time spent ruminating/DMI), both expressed in minutes per kilogram of DM. Moreover, the eating rate of NDF (ERNDF) was calculated by dividing the time spent eating by NDF intake, and the rumination rate of NDF (RRNDF) was determined by dividing the time spent ruminating by NDF intake. Both ERNDF and RRNDF were expressed in minutes per kilogram of NDF.

Samples of diets and orts were also collected for the determination of particle size distribution, which was performed by sieving using the Penn State Particle Size Separator and reported on an as-fed basis as described by Heinrichs and Kononoff (14). Particle sorting was determined as follows: *n* intake / *n* predicted intake, in which *n* = particle fraction retained on screens of 19 mm (long), 8 mm (medium), and 1.18 mm (short) and a pan (fine). Particle sorting values equal to 1 indicate no sorting. Those <1 indicate selective refusal (sorting against), and those >1 indicate preferential consumption [sorting for (22)].

### Liver Abscess, Rumen, and Cecum Morphometrics

At the end of adaptation, one animal per pen (*n* = 30) was slaughtered for evaluation of liver abscesses, rumenitis, rumen morphometrics, and cecum lesions. The remaining 90 Nellore yearling bulls were harvested only at the end of the study, after 111 days on feed, and those bulls were also evaluated for liver abscesses, rumenitis, rumen morphometrics, and cecum lesions. Regarding liver abscesses, they were classified according to incidence according to Brink et al. (23). Rumenitis evaluation was performed after cattle evisceration, and all rumens were scored after washing. Rumen epithelium was classified according to the incidence of lesions (rumenitis) and abnormalities (e.g., papillae clumped) as described by Bigham and McManus (24) using a scale of 0 (no lesions and abnormalities noted) to 10 (severe ulcerative lesions). All rumens were scored by two trained individuals who were blinded to the treatments, and final data represent the average of the two scores.

A 1-cm^2^ fragment of each rumen was collected from the dorsal cranial sac and placed in a phosphate-buffered saline solution for future macroscopic morphometric measurements (25). The number of papillae per square centimeter of rumen wall (NOP) was determined manually. Twelve papillae were randomly collected from each fragment and scanned, and the mean papillae area (MPA) was determined using an image analysis system (Image Tool, version 2.01 alpha 4, UTHSCSA Dental Diagnostic Science, San Antonio, TX). The rumen wall absorptive surface area (ASA), in square centimeters, was calculated as follows: 1 + (NOP × MPA) – (NOP × 0.002), where 1 represents the 1-cm^2^ fragment collected and 0.002 is the estimated basal area of papillae in square centimeters. The papillae area, expressed as a percentage of ASA, was calculated as follows: (NOP × MPA)/ASA × 100.

A 1-cm^2^ fragment of each rumen was likewise collected from the ventral cranial sac for microscopic morphometric measurements. Histological sections were stained with hematoxylin and eosin, embedded in paraffin wax, and sectioned (26). Histological measurements, such as papillae height, papillae width, papillae surface area, mitotic index related to the nuclei of the basal layer, and keratinized layer thickness were performed in 10% of ruminal papillae per animal (based on the NOP described earlier) using a computer-aided light microscope image analysis.

Cecum lesions evaluation was performed after cattle evisceration, and all washed ceca were scored. The cecum epithelium was classified according to the presence of cecal wall inflammation, lesions, and petechiae using a scale of 0 (no lesions noted) to 10 (severe lesions), as adapted from Bigham and McManus (24). All ceca were scored by two trained individuals who were blinded to the treatments, and final data represented the average of the two scores. A 1-cm^2^ fragment was collected from the center of each cecum epithelium for histological assessment and preserved in buffered paraformaldehyde 4% solution until future histological analyses (27). For the histological analysis, tissue samples were dehydrated and embedded in paraffin wax, sectioned at 8 μm, and stained with hematoxylin and eosin. Histological measurements, such as crypt depth and goblet cells, were determined in 10% of the total number of crypts per animal using a Leica Qwin Image Analyzer within a Leica electron light microscope.

### Apparent Total Tract Starch Digestibility

One animal per pen was randomly chosen to represent the pen during all collection periods. The feed and feces samples were collected 2 days after the end of the adaptation period as follows: VM6: collections on days 8, 9, and 10; VM9: collections on days 11, 12, and 13; and VM14, MON14, and MONVM14: collections on days 16, 17, and 18 of the experimental periods. The samples were collected once a day in the morning (06:00 h). In order to not disrupt their natural behavior, the samples were collected from the ground after the animals defecated, placed in a plastic bag, and put in a freezer at −20°C for future analysis. Similarly, in the finishing period, the samples were collected on days 69, 70, and 71 of the study following the same procedures just described.

The determination of starch content in samples of diets and feces was performed according to Pereira (28) and Hendrix (29). Moreover, the determination of dietary and fecal protein was performed by the Kjeldahl method according to Silva (30), and then starch digestibility was calculated (31).

### Statistical Analysis

The experimental design was a completely randomized block, and the initial BW was utilized as a criterion for block formation. Pens were considered the experimental unit for this study (*n* = 30), and each treatment was replicated six times. Data on feedlot performance, feeding behavior, particle sorting, starch digestibility, and carcass characteristics were analyzed using the MIXED procedure of SAS (SAS Inst., Inc., Cary, NC), in which the model included the effects of treatments as fixed and of block as random. The CONTRAST option of SAS was used to create contrasts in order to assess the following effects and comparisons: (1) linear relationship between days of adaptation when only VM was fed (6, 9, and 14 days) and the dependent variable, (2) quadratic relationship between days of adaptation when only VM was fed (6, 9, and 14 days) and the dependent variable, (3) MON14 vs. VM14, and (4) MONVM14 vs. VM14. As the days of adaptation were unequally spaced, we used a SAS macro (ORPOLY), which finds contrast coefficients for orthogonal polynomials for testing a quantitative factor variable and constructs CONTRAST statements using these values.

Data on rumen and cecum morphometrics were analyzed including the CONTRAST option, as just described, as repeated measures using the MIXED procedure of SAS and Tukey test to compare means. Period was included as the REPEATED option of SAS, with pen as the subject. The model included the effects of treatments, period (adaptation and finishing), and treatments × period interaction. As described earlier, block was included in the model as a random effect. Each variable analyzed as repeated measures was subjected to eight covariance structures: unstructured, compound symmetric, heterogeneous compound symmetric, autoregressive of order one, heterogeneous first-order autoregressive, toeplitz, heterogeneous toeplitz, and ante-dependence of order one. The covariance structure that yielded the smaller Akaike and Schwarz's Bayesian criterion based on their −2 res log likelihood was considered to provide the best fit. Results were considered significant at *P* ≤ 0.05 level. After the distribution of residuals, tests for normality (Shapiro–Wilk and Kolmogorov–Smirnov) and heterogeneity of treatment variances (GROUP option of SAS) were performed before analyzing the data.

## Results

### Feedlot Performance

The results of feedlot performance are presented in Table 2. No effects of treatments were observed (*P* < 0.10) for BW and ADG overall; however, cattle fed MON14 had greater (*P* = 0.05) ADG than those fed VM14 on the first half of the study (1.35 vs. 1.14 kg). During the first 28 days on feed, the DMI decreased linearly as the adaptation was shortened (*P* = 0.01) for the cattle fed VM as a sole feed additive. Furthermore, the cattle fed VM14 had a greater (*P* ≤ 0.03) DMI (9.21 kg) than those fed either MON14 (8.67 kg) or MONVM14 (8.60 kg) from day 1 to 28. In addition, the cattle fed VM14 presented a greater (*P* ≤ 0.03) DMI, expressed as % of BW, throughout the study when compared to the cattle fed either MON14 or MONVM14. Overall, the DMI was affected quadratically (*P* ≤ 0.02) for the cattle receiving only VM as source of feed additive, where animals adapted for 9 days presented the greatest intakes. Regarding feed efficiency after 111 days on feed, the cattle fed either MON14 or MONVM14 had an improved G/F by 10.4 or 8.1%, respectively, when compared to the bulls fed VM14. In addition, the cattle receiving MON14 also presented an improved G/F when compared to those fed VM14 on the first 28 days on feed (*P* = 0.05). No linear or quadratic VM effect (*P* > 0.35) was observed for G/F in this study.

**Table 2:** Feedlot performance of Nellore yearling bulls consuming high-concentrate diets containing sodium monensin (MON), virginiamycin (VM), or both during adaptation and finishing periods.

**Item**	**Treatments** ^**a**^					**SEM**	***P*** **-value**			
	**MON**	**MON** **+ VM**		**VM**			**MON14** **vs. VM14**	**MONVM14** **vs. VM14**	**VM effect** ^**b**^	
	**14**	**14**	**6**	**9**	**14**				**L**	**Q**
Initial BW, kg	390.15	390.75	390.18	390.33	390.65	7.61	0.82	0.97	0.91	0.97
BW at day 28	425.28	420.69	417.76	419.36	420.90	8.53	0.38	0.97	0.52	0.99
BW at day 56	466.83	457.81	458.67	462.51	455.52	9.12	0.11	0.74	0.65	0.37
BW at day 84	509.49	506.67	502.51	508.00	503.63	9.49	0.34	0.62	0.86	0.36
Final BW, kg	552.62	549.08	543.65	548.13	542.08	10.73	0.16	0.35	0.83	0.41
ADG, kg										
Days 1–28	1.21	1.03	0.95	1.00	1.04	0.10	0.27	0.94	0.53	0.97
Days 1–56	1.35	1.18	1.21	1.27	1.14	0.07	0.05	0.70	0.53	0.28
Days 1–84	1.40	1.37	1.33	1.39	1.33	0.05	0.27	0.59	0.91	0.30
Days 1–111	1.45	1.42	1.37	1.41	1.35	0.05	0.12	0.31	0.76	0.36
Daily DMI, kg										
Days 1–28	8.67	8.60	8.59	9.06	9.21	0.22	0.03	0.02	0.01	0.47
Days 1–56	9.17	9.03	9.35	9.84	9.50	0.22	0.15	0.04	0.49	0.04
Days 1–84	9.59	9.58	9.84	10.32	9.91	0.22	0.16	0.16	0.76	0.03
Days 1–111	9.74	9.70	9.92	10.43	10.03	0.22	0.20	0.15	0.63	0.02
Daily DMI, % of BW										
Days 1–28	2.13	2.12	2.12	2.23	2.27	0.03	0.01	<0.01	<0.01	0.36
Days 1–56	2.16	2.14	2.22	2.33	2.26	0.03	0.01	<0.01	0.33	<0.01
Days 1–84	2.14	2.16	2.23	2.32	2.24	0.03	0.01	0.03	0.61	<0.01
Days 1–111	2.11	2.12	2.18	2.28	2.21	0.03	0.01	0.03	0.47	<0.01
Gain-to-feed ratio										
Days 1–28	0.140	0.120	0.110	0.110	0.112	0.011	0.05	0.61	0.89	0.94
Days 1–56	0.147	0.130	0.129	0.129	0.120	0.006	<0.01	0.27	0.35	0.54
Days 1–84	0.147	0.143	0.135	0.135	0.134	0.004	0.02	0.10	0.94	0.99
Days 1–111	0.149	0.146	0.138	0.135	0.135	0.003	<0.01	0.02	0.46	0.73
DMI fluctuation, %										
Days 1–28	8.04	8.85	10.87	9.66	8.31	0.76	0.79	0.59	0.02	0.93
Days 1–56	6.23	7.44	8.74	7.56	7.99	0.42	0.01	0.36	0.21	0.13
Days 1–84	5.56	6.35	7.12	6.49	7.10	0.35	<0.01	0.14	0.97	0.16
Days 1–111	5.35	6.16	6.95	6.21	6.78	0.35	<0.01	0.22	0.73	0.13
DMI fluctuation, kg										
Days 1–28	0.67	0.68	0.84	0.82	0.71	0.06	0.63	0.72	0.05	0.41
Days 1–56	0.55	0.63	0.75	0.71	0.74	0.03	<0.01	0.02	0.69	0.32
Days 1–84	0.52	0.58	0.66	0.65	0.69	0.03	<0.01	0.03	0.49	0.53
Days 1–111	0.74	0.81	0.88	0.88	0.90	0.04	<0.01	0.11	0.76	0.79
Cost to gain a kilogram of BW, R$	6.89	7.13	7.50	7.63	7.70	0.19	<0.01	0.02	0.41	0.88
NEm, Mcal/kg of DM	2.08	2.06	1.97	1.93	1.94	0.03	<0.01	<0.01	0.40	0.42
Neg, Mcal/kg of DM	1.34	1.38	1.38	1.40	1.32	0.31	0.62	0.23	0.18	0.28
NEm/ NEm expected	0.99	0.98	0.92	0.91	0.92	0.16	<0.01	<0.01	0.78	0.67
NEg/NEg expected	1.14	1.12	1.06	1.03	1.04	0.02	<0.01	<0.01	0.58	0.36

*BW, body weight; ADG, average daily gain; DMI, dry matter intake; NEm, net energy for maintenance; Neg, net energy for gain; SEM, standard error of mean*.

a *MON14, 27mg of MON/kg of DM and adaptation for 14 days; MON + VM14, 27mg of MON/kg of DM and 25mg of VM/kg of DM and adaptation for 14 days; VM14, 25mg of VM/kg of DM and adaptation for 14 days; VM9, 25mg of VM/kg of DM and adaptation for 9 days; VM6, 25mg of VM/kg of DM and adaptation for 6 days*.

b *Linear (L) and quadratic (Q) responses for the effect of adaptation length in cattle fed only VM*.

For the first 28 days on feed, the DMI fluctuation, expressed both in percent and kilogram, increased linearly (*P* ≤ 0.05; Table 2) by shortening the adaptation period from 14 to 6 days for the cattle fed VM as the sole feed additive. No further differences were detected (*P* > 0.13) for DMI fluctuations in the cattle fed only VM for the rest of the study. Nevertheless, when compared to the cattle receiving MON, the animals fed VM14 had a greater (*P* < 0.01) DMI fluctuation than those consuming MON14 overall, as well as a greater (*P* = 0.03) fluctuation in percent than the cattle consuming MONVM14 from 1 to 84 days on feed. The feeding of MON14 decreased DMI fluctuation throughout the study by 21 and 18%, when expressed in percent and kilogram, respectively, when compared to the cattle consuming VM14.

No VM effect was detected (*P* > 0.18) for any variables evaluated that were related to net energy in this study. In addition, no VM effect was observed (*P* > 0.41) on the cost to gain a kilogram of BW. On the other hand, the cattle fed MON14 and MONVM14 had a decreased cost to gain a kilogram of BW by 10.5% (*P* < 0.01) and 7.4% (*P* = 0.02), respectively, when compared to those fed VM14. Furthermore, the animals consuming MON14 and MONVM14 had increased (*P* < 0.01) diet NEm and NEg in relation to the expected when compared to the cattle fed VM14.

### Feeding Behavior and Particle Sorting

The results on feeding behavior evaluated right after the end of the adaptation period are summarized in Table 3. No significant effects of treatments were observed (*P* < 0.10) for time spent resting, ruminating, and eating, as well as for meals per day and ERDM. On the other hand, as the adaptation period was shortened for the cattle fed only VM, DMI (*P* = 0.01), and DMI per meal (*P* < 0.01) linearly decreased (*P* ≤ 0.01). In addition, the cattle fed VM14 had a greater DMI per meal than the animals consuming either MON14 (*P* = 0.01) or MONVM14 (*P* = 0.05). Moreover, the RRDM increased linearly (*P* = 0.02) by shortening the adaptation for those cattle fed VM as the sole feed additive. The meal length and NDF intake were affected quadratically (*P* ≤ 0.05) as the adaptation period was shortened for the cattle receiving only VM, where those animals consuming VM9 presented a greater NDF intake, leading to a longer meal length. As a result, a quadratic response was also observed for ERNDF (*P* < 0.01) and RRNDF (*P* = 0.03), in which the cattle fed VM9 showed a decreased ERNDF and RRNDF.

**Table 3:** Feeding behavior and particle sorting of Nellore yearling bulls consuming high-concentrate diets containing sodium monensin (MON), virginiamycin (VM), or both right after the end of the adaptation period (day 7, 10, or 15).

**Item**	**Treatments** ^**a**^					**SEM**	***P*** **-value**			
	**MON**	**MON + VM**		**VM**			**MON14 vs. VM14**	**MONVM14 vs. VM14**	**VM effect** ^**b**^	
	**14**	**14**	**6**	**9**	**14**				**L**	**Q**
**Feeding behavior**										
Time spent resting, min	926.4	910	925.6	932.2	921.7	21.8	0.88	0.71	0.90	0.75
Time spent ruminating, min	303.9	318.3	331.7	318.3	324.4	18.0	0.42	0.81	0.78	0.66
Time spent eating, min	209.7	211.7	182.8	189.4	193.9	12.0	0.36	0.38	0.58	0.95
Meal length, min	15.9	16.8	24.0	30.4	19.5	3.9	0.50	0.62	0.40	0.05
Meals per day, *n*	5.4	4.6	3.06	2.7	3.8	0.7	0.13	0.47	0.44	0.37
DMI, kg	8.8	9.3	7.1	7.4	9.5	0.6	0.47	0.78	0.01	0.23
DMI per meal, kg	0.67	0.75	0.56	0.66	0.98	0.08	0.01	0.05	<0.01	0.29
ERDM, min/kg of DM	23.8	23.1	25.9	31.0	20.9	3.73	0.58	0.68	0.34	0.11
RRDM, min/kg of DM	34.4	34.5	47.8	48.5	34.9	4.13	0.93	0.94	0.02	0.12
NDF intake, kg	2.35	2.46	2.06	3.16	2.30	0.24	0.86	0.57	0.47	<0.01
ERNDF, min/kg of NDF	91.5	87.6	97.5	61.8	117.6	7.6	0.70	0.99	0.34	<0.01
RRNDF, min/kg of NDF	133.5	131.7	189.6	107.4	144.3	15.9	0.42	0.16	0.30	0.03
**Particle sorting**										
Long	0.89	0.69	1.11	1.19	0.99	0.10	0.41	0.04	0.41	0.28
Medium	0.98	0.92	1.14	1.18	1.03	0.04	0.34	0.05	0.05	0.05
Short	1.01	1.02	1.01	1.02	1.02	0.01	0.38	0.85	0.65	0.93
Fine	0.99	1.00	0.96	0.81	0.95	0.05	0.52	0.49	0.86	0.02

*DMI, dry matter intake; ERDM, eating rate of dry matter; RRDM, rumination rate of dry matter; NDF, neutral detergent fiber; ERNDF, eating rate of NDF; RRNDF, rumination rate of NDF; SEM, standard error of mean*.

a *MON14, 27mg of MON/kg of DM and adaptation for 14 days; MON + VM14, 27mg of MON/kg of DM and 25mg of VM/kg of DM and adaptation for 14 days; VM14, 25mg of VM/kg of DM and adaptation for 14 days; VM9, 25mg of VM/kg of DM and adaptation for 9 days; VM6, 25mg of VM/kg of DM and adaptation for 6 days*.

b *Linear (L) and quadratic (Q) responses for the effect of adaptation length in cattle fed only VM*.

In terms of particle sorting, the results obtained right after the end of the adaptation period are shown in Table 3. No significant VM effect (*P* > 0.28) was detected for sorting of long and short particles; however, the cattle consuming MONVM14 sorted significantly (*P* = 0.04) against long particles when compared to those fed VM14. A significant quadratic response was likewise observed for the sorting of medium (*P* = 0.05) and fine particles (*P* = 0.02) in the cattle fed only VM, where animals consuming VM9 sorted more intensively for medium and against fine particles. In addition, the cattle receiving MONVM14 sorted significantly (*P* = 0.05) against medium particles when compared to the animals consuming VM14.

The results on feeding behavior evaluated on day 60 of the study, during the finishing period, are shown in Table 4. No significant treatment effect was observed (*P* > 0.10) for the feeding behavior variables evaluated except for time spent ruminating, meals per day, and DMI per meal. The bulls fed MON14 spent less time ruminating and had a lesser DMI per meal than those fed VM14 (*P* ≤ 0.05). The cattle consuming MONVM14 also presented a lesser (*P* = 0.05) DMI per meal when compared to the animals fed VM14. Furthermore, the DMI per meal decreased linearly (*P* = 0.05) as the adaptation period was shortened for the cattle fed only VM. In addition, the shortening of the adaptation period for the animals consuming only VM impacted the meals per day quadratically (*P* = 0.05), where the cattle receiving VM9 attended bunks more frequently, resulting in a higher number of daily meals.

**Table 4:** Feeding behavior and particle sorting of Nellore yearling bulls consuming high-concentrate diets containing sodium monensin (MON), virginiamycin (VM), or both in the finishing period (after 60 days on feed).

**Item**	**Treatments** ^**a**^					**SEM**	***P*** **-value**			
	**MON**	**MON + VM**	**VM**				**MON1** **vs. VM14**	**MONVM14 vs. VM14**	**VM effect** ^**b**^	
	**14**	**14**	**6**	**9**	**14**				**L**	**Q**
**Feeding behavior**										
Time spent resting, min	970.8	935.6	966.7	945.6	939.2	16.9	0.18	0.87	0.24	0.71
Time spent ruminating, min	293.1	313.9	306.9	238.3	333.3	14.4	0.03	0.28	0.15	0.60
Time spent eating, min	176.1	190.6	166.4	66.1	167.5	10.3	0.56	0.12	0.93	0.95
Meal length, min	16.4	17.4	15.2	14.5	17.5	1.0	0.46	0.95	0.11	0.14
Meals per day, *n*	10.8	11	10.9	11.8	9.7	0.6	0.20	0.15	0.17	0.05
DMI, kg	10.0	10.3	10.1	10.4	10.4	0.4	0.45	0.78	0.62	0.87
DMI per meal, kg	0.94	0.93	0.94	0.91	1.10	0.10	0.05	0.05	0.05	0.17
ERDM, min/kg of DM	17.8	18.7	16.3	16.1	16.3	1.1	0.31	0.10	0.97	0.84
RRDM, min/kg of DM	29.5	30.8	30.5	32.1	32.6	2.0	0.26	0.51	0.44	0.80
NDF intake, kg	2.73	2.90	2.53	2.53	3.16	0.30	0.33	0.55	0.14	0.38
ERNDF, min/kg of NDF	69.0	69.3	66.3	72.9	58.3	7.8	0.30	0.30	0.43	0.26
RRNDF, min/kg of NDF	119.4	114.3	124.5	141.3	113.4	15.6	0.79	0.97	0.62	0.25
**Particle sorting**										
Long	1.03	0.96	1.03	1.00	1.02	0.03	0.74	0.27	0.72	0.53
Medium	0.96	0.99	1.03	1.10	1.02	0.03	0.31	0.67	0.72	0.05
Short	1.01	1.01	1.01	1.01	1.01	<0.01	0.76	0.98	0.63	0.89
Fine	0.98	0.99	0.96	0.89	0.98	0.04	0.97	0.91	0.70	0.05

*DMI, dry matter intake; ERDM, eating rate; RRDM, rumination rate; NDF, neutral detergent fiber; ERNDF, eating rate of NDF; RRNDF, rumination rate of NDF; SEM, standard error of mean*.

a *MON14, 27mg of MON/kg of DM and adaptation for 14 days; MON + VM14, 27mg of MON/kg of DM and 25mg of VM/kg of DM and adaptation for 14 days; VM14, 25mg of VM/kg of DM and adaptation for 14 days; VM9, 25mg of VM/kg of DM and adaptation for 9 days; VM6, 25mg of VM/kg of DM and adaptation for 6 days*.

b *Linear (L) and quadratic (Q) responses for the effect of adaptation length in cattle fed only VM*.

With respect to particle sorting evaluated on day 60 of the study, during the finishing period, the results are shown in Table 4. No significant effect of treatments (*P* > 0.27) was detected for sorting of long and short particles; however, a significant quadratic response was observed for the sorting of medium (*P* = 0.05) and fine particles (*P* = 0.05) in the cattle fed only VM, where animals consuming VM9 kept sorting more intensively for medium and against fine particles.

### Carcass Traits

The results related to the carcass traits of Nellore yearling bulls are presented in Table 5. No treatment main effect was observed (*P* > 0.05) for most of the carcass trait variables evaluated; however, the shortening of the adaptation period led to a linear decrease of the 12th rib fat (*P* = 0.02) and BF fat daily gain (*P* = 0.02) of the Nellore bulls fed only VM, which linearly decreased the final BF fat thickness (*P* < 0.01) in those animals. Furthermore, the bulls fed VM14 increased the final BF thickness by 14.3% when compared to the cattle fed MON14 (8.48 vs. 7.42 mm; *P* = 0.03). In addition, for the cattle fed VM as the sole feed additive, the LM area daily gain was affected quadratically (*P* = 0.05) as the adaptation period was shortened, where the cattle fed VM9 had a lower LM tissue accretion.

**Table 5:** Carcass traits of Nellore yearling bulls consuming high-concentrate diets containing sodium monensin (MON), virginiamycin (VM), or both during the adaptation and finishing periods.

**Item**	**Treatments** ^**a**^					**SEM**	***P*** **-value**			
	**MON**	**MON + VM**		**VM**			**MON14** **vs. VM14**	**MONVM14 vs. VM14**	**VM effect** ^**b**^	
	**14**	**14**	**6**	**9**	**14**				**L**	**Q**
HCW, kg	303.06	295.28	298.33	300.28	296.61	6.43	0.21	0.79	0.74	0.53
Dressing, %	54.86	53.74	54.89	54.80	54.97	0.29	0.80	0.01	0.86	0.71
Initial LM3 area, cm^2^	65.09	64.63	66.73	67.61	68.02	1.27	0.21	0.18	0.44	0.87
Final LM area, cm^2^	79.45	78.51	80.77	78.41	80.64	1.92	0.65	0.43	0.96	0.32
LM area daily gain, cm^2^	0.128	0.124	0.125	0.096	0.113	0.013	0.32	0.47	0.41	0.05
Initial 12th rib fat, mm	2.37	2.60	2.39	2.43	2.58	0.10	0.14	0.88	0.19	0.63
Final 12th rib fat, mm	5.61	6.26	4.87	5.60	5.99	0.25	0.51	0.70	0.08	0.56
12th rib fat daily gain, mm	0.029	0.033	0.022	0.028	0.031	0.002	0.66	0.51	0.02	0.48
Initial BF fat thickness, mm	3.39	3.84	3.46	3.52	3.84	0.12	0.01	0.98	0.03	0.35
Final BF fat thickness, mm	7.42	8.58	7.07	8.16	8.48	0.33	0.03	0.82	<0.01	0.34
BF fat daily gain, mm	0.036	0.042	0.032	0.041	0.041	0.003	0.16	0.80	0.02	0.17
Initial marbling	1.94	2.16	2.12	1.97	2.02	0.12	0.66	0.40	0.54	0.49
Final marbling	2.81	3.06	2.84	2.63	2.63	0.11	0.29	0.02	0.21	0.45

*HCW, hot carcass weight; LM, longissimus muscle; BF, biceps femoris muscle; SEM, standard error of mean*.

a *MON14, 27mg of MON/kg of DM and adaptation for 14 days; MON + VM14, 27mg of MON/kg of DM and 25mg of VM/kg of DM and adaptation for 14 days; VM14, 25mg of VM/kg of DM and adaptation for 14 days; VM9, 25mg of VM/kg of DM and adaptation for 9 days; VM6, 25mg of VM/kg of DM and adaptation for 6 days*.

b *Linear (L) and quadratic (Q) responses for the effect of adaptation length in cattle fed only VM*.

The cattle fed VM14 increased (*P* = 0.01) the dressing percentage by 1.23% but had a lower (*P* = 0.02) marbling score at the end of the study when compared to the Nellore bulls fed MONVM14.

### Liver Abscess, Rumen, and Cecum Morphometrics

No liver abscess was found in the animals evaluated in this study. The results related to rumen and cecum morphometrics are presented in Table 6. The shortening of the adaptation period for the cattle fed only VM did not negatively impact the rumenitis score (*P* > 0.19). However, the cattle receiving VM14 presented a higher (*P* < 0.01) incidence of rumen lesions when compared to those fed MONVN14 (0.85 vs. 0.38).

**Table 6:** Rumen and cecum morphometrics of Nellore yearling bulls consuming high-concentrate diets containing sodium monensin (MON), virginiamycin (VM), or both during the adaptation and finishing periods.

**Item**	**Treatments** ^**a**^							**SEM**	***P*** **-value**				
	**MON**	**MON + VM**		**VM**		**Period**			**MON14 *vs*. VM14**	**MONVM14 *vs*. VM14**	**VM effect** ^**b**^		**Period**
	**14**	**14**	**6**	**9**	**14**	**ADAP**	**TERM**				**L**	**Q**	
Rumenitis score	0.86	0.38	0.96	1.09	0.85	0.59	1.06	0.83	0.97	<0.01	0.50	0.19	<0.01
**Macroscopic variables**													
Mean papillae area, cm^2^	0.42	0.40	0.36	0.32	0.34	0.36	0.38	0.37	<0.01	0.01	0.37	0.05	0.59
ASA, cm^2^/cm^2^ of rumen wall	33.8	29.5	35.9	25.6	28.9	30.7	30.7	1.9	0.05	0.82	0.01	<0.01	0.99
Number of papillae, n	80.3	74.6	99.4	78.7	88.2	84.8	83.7	5.7	0.26	0.08	0.33	0.04	0.85
Papillae area, % of ASA	97.3	96.9	97.5	96.5	97.0	97.1	97.0	0.2	0.20	0.72	0.07	<0.01	0.86
**Microscopic variables**													
Papillae height, mm	3.13	3.00	3.04	3.00	3.41	2.62	3.60	0.28	0.48	0.28	0.36	0.48	<0.01
Papillae width, mm	0.28	0.35	0.37	0.35	0.38	0.30	0.37	0.02	<0.01	0.20	0.71	0.18	0.08
Papillae surface area, mm^2^	1.19	1.07	1.24	1.03	1.27	0.95	1.37	0.18	0.80	0.42	0.94	0.33	<0.01
Keratinized layer thickness, μm	9.6	10.9	11.0	10.9	11.6	10.3	11.4	0.5	0.01	0.34	0.43	0.52	0.14
Mitotic index, %	3.2	2.9	3.3	3.1	3.1	3.1	3.1	0.1	0.92	0.14	0.50	0.65	0.93
Mitotic index, *n*	63.2	58.2	65.1	62.8	62.9	62.4	62.5	2.18	0.93	0.14	0.50	0.65	0.93
**Cecum morphometrics**													
Cecum lesions score^c^	1.1	1.9	0.9	1.3	2.1	0.8	2.1	0.4	0.11	0.72	0.05	0.73	<0.01
Crypt depth, μm	121.9	154.0	161.2	145.4	158.9	148.7	147.8	11.7	0.01	0.73	0.87	0.23	0.93
Goblet cells, *n*	40.7	38.1	33.5	39.1	35.0	38.8	35.7	1.3	<0.01	0.31	0.34	0.01	0.08
Crypt depth/goblet cells	3.2	4.5	5.0	3.8	4.8	4.2	4.3	0.5	0.01	0.55	0.80	0.05	0.80

*ASA, absorptive surface area; SEM, standard error of mean*.

a *MON14, 27mg of MON/kg of DM and adaptation for 14 days; MON + VM14, 27mg of MON/kg of DM and 25mg of VM/kg of DM and adaptation for 14 days; VM14, 25mg of VM/kg of DM and adaptation for 14 days; VM9, 25mg of VM/kg of DM and adaptation for 9 days; VM6, 25mg of VM/kg of DM and adaptation for 6 days*.

b *Linear (L) and quadratic (Q) responses for the effect of adaptation length in cattle fed only VM*.

c *Interaction between treatments and periods (P = 0.03)*.

Regarding the rumen macroscopic variables evaluated, MPA, ASA, NOP, and papillae area as percent of ASA of the bulls fed only VM were affected quadratically (*P* ≤ 0.05) as the adaptation period was shortened from 14 to 6 days, where the cattle fed VM9 had a lower NOP and a smaller MPA, ASA, and papillae area expressed as percent of ASA. Furthermore, the cattle fed VM14 had a smaller MPA (*P* ≤ 0.01) when compared to the animals fed either MON14 or MONVM14, as well as a smaller (*P* < 0.05) ASA than the cattle receiving MON14 (28.9 vs. 33.8 cm^2^).

With respect to rumen microscopic variables, no VM effect was observed (*P* > 0.18); however, the cattle fed VM14 had greater papillae width (*P* < 0.01) and keratinized layer thickness (*P* = 0.01) than the bulls consuming MON14.

**Figure 1:**
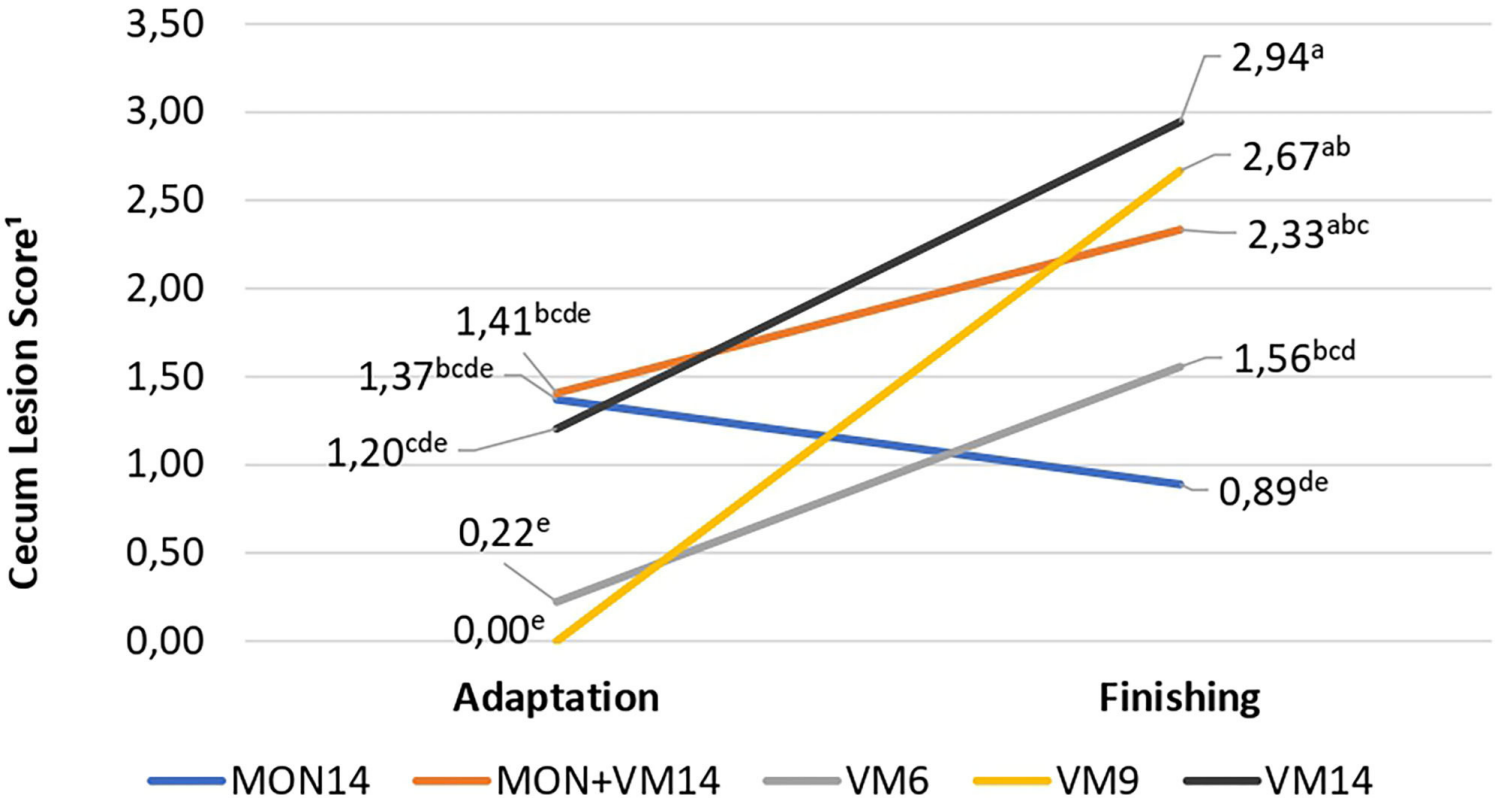
Interaction of treatments with periods (adaptation and finishing) for cecum lesions score of Nellore cattle consuming high-concentrate diets containing sodium monensin (MON), virginiamycin (VM), or both during adaptation and finishing period. MON14, 27mg of MON/kg of dry matter (DM) and adaption for 14 days; MON + VM14, 27mg of MON/kg of DM and 25mg of VM/kg of DM and adaption for 14 days; VM14, 25mg of VM/kg of DM and adaption for 14 days; VM9, 25mg of VM/kg of DM and adaption for 9 days; and VM6, 25mg of VM/kg of DM and adaption for 6 days. Means without a common superscript letter differ (*P* < 0.05); pooled SEM (0.59 and 0.43).

In terms of cecum morphometrics, an interaction of treatments with periods (adaptation and finishing) was observed (*P* = 0.03), where no differences across treatments were detected at the end of the adaptation period; however, the cattle fed VM14 and VM9 had higher cecum scores than the bulls receiving MON14 (Figure 1) at the end of the finishing period. The animals fed only VM, regardless of adaptation in 6, 9, or 14 days, increased the cecum scores from adaptation to finishing, which did not occur with those consuming MON. For crypt depth, the cattle fed VM14 had deeper (*P* = 0.01) crypts than those fed MON14. In addition, a quadratic VM effect was observed for the number of goblet cells (*P* = 0.01) and crypt depth/goblet cells ratio (*P* = 0.05), where the cattle fed VM9 had lower numbers of goblet cells and a greater crypt depth/goblet cells ratio.

### Apparent Total Tract Starch Digestibility

The results on apparent total tract starch digestibility are presented in Table 7. No treatment effect was observed (*P* > 0.10) for fecal starch and nitrogen percentage as well as for starch digestibility either in the adaptation or in the finishing period.

**Table 7:** Fecal starch and apparent total tract digestibility of starch of Nellore yearling bulls consuming high-concentrate diets containing sodium monensin (MON), virginiamycin (VM), or both during the adaptation and finishing periods.

**Item**	**Treatments** ^**a**^					**SEM**	***P*** **-value**			
	**MON**	**MON + VM**		**VM**			**MON14 vs. VM14**	**MONVM14 vs. VM14**	**VM effect** ^**b**^	
	**14**	**14**	**6**	**9**	**14**				**L**	**Q**
**Adaptation**										
Fecal starch, g/kg	74.3	87.5	98.0	102	106	19	0.26	0.51	0.78	0.99
Fecal nitrogen, g/kg	20	20	20	19	19	0.8	0.50	0.27	0.47	0.83
Digestibility, g/kg	943	931	923	908	926	19	0.52	0.87	0.91	0.48
**Finishing**										
Fecal starch, g/kg	139	131	122	112	109	17	0.31	0.21	0.41	0.91
Fecal nitrogen, g/kg	20	21	22	21	20	0.5	0.72	0.60	0.12	0.81
Digestibility, g/kg	906	918	926	928	932	13	0.17	0.47	0.75	0.96

*SEM, standard error of mean*.

a *MON14, 27mg of MON/kg of DM and adaptation for 14 days; MON + VM14, 27mg of MON/kg of DM and 25mg of VM/kg of DM and adaptation for 14 days; VM14, 25mg of VM/kg of DM and adaptation for 14 days; VM9, 25mg of VM/kg of DM and adaptation for 9 days; VM6, 25mg of VM/kg of DM and adaptation for 6 days*.

b *Linear (L) and quadratic (Q) responses for the effect of adaptation length in cattle fed only VM*.

## Discussion

### Feed Additives vs. Adaptation for 14 Days

It is well-reported in the literature that the Nellore cattle fed MON can be safely adapted to high-concentrate diets for periods not shorter than 14 days (3–5). Therefore, this section aims to discuss the potential of adapting Nellore cattle to high-concentrate diets in 14 days using VM as the sole feed additive. The cattle fed VM14 showed a greater DMI per meal during feeding behavior evaluations on both adaptation and finishing periods, which explains the greater DMI throughout the study, expressed either in kilograms or as percent of BW, when compared to the cattle fed MON14 or MONVM14 since no differences in meals per day or meal length were detected. It is documented in the literature (7, 8) that the negative effect of MON on DMI is related to a lower DMI per meal. As a result, the cattle consuming VM14 increased the DMI fluctuations by 22% (0.90 vs. 0.74 kg; Table 2) throughout the study in relation to the cattle fed MON14; however, the average daily fluctuation, expressed in percent, was 6.78 and 5.35% for the cattle fed VM14 and MON14, respectively. Other authors (32) reported that the performance is negatively impacted only when feedlot cattle present a DMI fluctuation higher than 10%. Therefore, no differences on final BW and ADG were detected when the cattle fed VM14, MON14, or MONVM14 were compared in this study (Table 2). Moreover, since the feeding of VM as the sole feed additive increased the DMI without positive impacts on ADG, the cattle fed VM14 had a feed efficiency negatively impacted by 9.4 and 7.5% when compared to the bulls fed MON14 and MONVM14, respectively. It has been previously reported that VM does not negatively impact DMI (8, 11) as MON does (7). As a result, the cattle fed VM14 increased the cost to gain a kilogram of BW by 11.8 and 8.0% in relation to animals receiving MON14 and MONVM14, respectively.

It is noteworthy to mention that the negative impact of MON on DMI, expressed as percent of BW, also negatively affected the final BF thickness when compared to the cattle receiving VM14. In fact, the cattle fed VM14 had an increased DMI, as percent of BW, by 4.7% when compared to the cattle consuming MON14 (Table 5), which may have contributed to greater fat deposition due to the increased amount of energy consumed throughout the study since no differences were observed for starch digestibility during adaptation or finishing (Table 7). This negative effect of MON on carcass fat deposition, as shown in this study, has been reported previously by other authors (33), and it is potentially related to the decreasing molar proportion of ruminal acetate (34). Therefore, the impaired feed efficiency presented by the cattle fed VM14 may be partially attributed to more extensive fat deposition, which requires more energy or calories per gram of tissue than muscle accretion (35, 36). The NEg/NEg expected ratio likewise improved by only 4% for the cattle fed VM14, whereas for the cattle receiving MON14 and MONVM14 it improved by 14 and 12%, respectively, without negative impacts on the HCW. In addition, the cattle fed MONVM14 sorted against long and medium particles during adaptation, which may explain the lesser DMI when compared to those fed VM14 and the negative impact on dressing percentage; however, this might also explain their improved marbling. Thus, when fat cover is a variable of economic importance to determine the carcass value, the use of VM as the sole feed additive may be an alternative for cattle adapted for 14 days.

With respect to the rumen variables evaluated, the cattle receiving VM14 had decreased MPA by 21% and ASA by 16% when compared to the cattle fed MON14, which may be associated to a more extensive increase of the molar proportion of ruminal propionate in the cattle fed MON (37). Costa et al. (38) reported that propionate is the short-chain fatty acid (SCFA) most responsible for promoting the metabolically active growth of ruminal papillae. Moreover, Melo et al. (39) reported that the ASA of the rumen wall was the morphometric variable most correlated to the speed of SCFA absorption. Based on facts just described, the cattle fed VM14 also had a smaller MPA than those animals fed MONVM14. Apparently, decreasing the ruminal acetate/propionate ratio in the cattle fed MON14 contributed to a larger development of rumen epithelium, which allows a faster SCFA clearance; however, carcass fat deposition was impaired. On the other hand, due to a greater DMI associated with a smaller ASA for SCFA absorption, the cattle fed VM14 had greater papillae width and keratinized layer thickness than the animals consuming MON14, which may be a consequence of a more extensive ruminal acidification. As a result, cattle consuming VM14 had higher rumenitis scores than animals receiving MONVM14, which is also a variable to explain ruminal acidification. Thus, it is clear that the cattle fed VM14 showed more evidence of ruminal acidification than those fed either MON14 or MONVM14 since keratinization and rumenitis are often detrimental to ruminal epithelium development and negatively impact ASA. It is noteworthy to mention that the cattle fed VM14 spent more time ruminating in the finishing than the animals fed MON14; however, it seems that ASA plays a more important role than rumination to increase the ruminal pH to adequate levels.

As a result, a greater amount of starch may have bypassed the rumen of the cattle fed VM14 and reached the intestines for digestion and absorption. In addition, part of this starch may have reached the cecum, which further developed their crypts, but not goblet cells at the same magnitude, when compared to the cattle fed MON14 (Table 7). Since no differences in fecal starch and total tract starch digestibility were detected, we assumed that the cecum was able to digest and absorb the SCFA that resulted from the ruminal bypass starch. However, the cattle fed VM14 had more lesions in the cecum than the animals consuming MON14 at the end of the study (Figure 1).

In summary, for the cattle adapted for 14 days, the use of VM as the sole feed additive in high-concentrate diets promoted similar ADG and HCW when compared to feeding only MON or the association of MON and VM; however, the greater DMI increased the DMI fluctuations and caused rumen lesions and keratinization as well as a higher incidence of lesions in the cecum. Thus, the lack of a negative effect on DMI when VM is fed impairs the G/F ratio and may compromise the adaptation of Nellore cattle to high-concentrate diets in periods shorter than 14 days.

### Use of VM to Shorten the Adaptation Period

The shortening of the adaptation period from 14 to 9 or 6 days did not negatively impact the feedlot performance overall, which includes variables such as final BW, ADG, G/F ratio, cost to gain a kilogram of BW, HCW, and dressing percentage. On the other hand, DMI (kg and %BW) responded quadratically as adaptation length was shortened, where the cattle fed VM9 had greater intakes.

However, the cattle fed VM9 sorted for medium and against fine diet particles during both the adaptation and finishing periods and consumed significantly more NDF during adaptation, which may be a response to control rumen acidification and a potential explanation for the increased DMI shown by the cattle consuming VM9. During the finishing period, the cattle fed VM9 also had more meals per day and less DMI intake per meal while trying to control rumen acidification. As a result, carcass fat deposition was negatively affected since shortening the adaptation period linearly decreased the 12th rib fat and BF fat daily gains. In addition, the cattle receiving VM9 presented the least LM area daily gain. Therefore, the cattle fed VM9 may have increased DMI overall to offset the lower amount of energy consumed. Despite being numerically greater, the rumenitis scores did not increase by shortening the adaptation period. Nevertheless, all macroscopic rumen variables of the cattle fed VM9 were negatively impacted, including MPA, ASA, NOP, and papillae area expressed as percent of ASA. Other authors (2) also evaluated the shortening of the adaptation period from 14 to 9 days for Nellore cattle and reported that caution is advised before adopting 9 days of adaptation since rumen papillae development was negatively impacted by the end of the adaptation, and no improvements in NOP and ASA and also in performance were detected at the end of the study. The same authors also calculated a cell proliferation index based on an immunohistochemistry technique performed on rumen papillae and reported that cattle adapted for 9 days had more nuclei proliferating at the end of the adaptation, which means that the rumen papillae were still adapting, but Nellore cattle adapted for 14 days had a cell proliferation index similar to the animals slaughtered at the end of the study. Since propionate is the SCFA most related to rumen epithelium development (38), the least energy intake throughout the study may have not properly stimulated propionate production in the cattle fed VM9, which may have led to smaller ASA and MPA. The fact that the cattle fed VM9 sorted against fine diet particles may have contributed to less starch intake; however, the chemical composition of the feed retained in each screen of the particle separator device was not determined in this study.

As a result, the lowest crypt depth/goblet cells ratio shown by the cattle fed VM9 may be associated to less starch granules available for fermentation in the cecum, which limited their cecum capacity to absorb SCFA, leading to the greatest increase on cecum lesions from the end of adaptation to the end of the finishing period in those cattle (Figure 1). Apparently, 9 days of adaptation to high-concentrate diets for Nellore cattle is not enough for them to achieve a properly developed rumen epithelium and avoid lesions in the cecum since the feeding behavior pattern is disrupted in an attempt to control rumen acidification. Authors from North America (6) suggested that cattle could not be adapted in less than 14 days without impairing feedlot performance and animal health. Such recommendation seems reasonable for Nellore cattle finished in feedlot as well, regardless of the diet energy content or the feed additive employed.

Finally, the cattle fed VM6 had increased DMI fluctuations during the first 28 days on feed, which included the adaptation period, even at a lower DMI, but no overall effect on DMI fluctuation was detected. The lowest DMI presented by the cattle fed VM6 from 0 to 28 days on feed may have helped them to cope with ruminal acidification; however, the cattle consuming VM6 still sorted for medium diet particles during adaptation as the VM9-fed cattle did. During finishing, the cattle fed VM6 also had more meals per day and less DMI intake per meal in relation to the cattle fed VM14. Previous studies have reported that 6 days of adaptation resulted in no improvement in feedlot performance and negative effects on rumen epithelium development (1, 4). As a result, despite the lack of VM effect on the HCW and ADG, the carcass fat deposition of the cattle fed VM6 was also negatively impacted by shortening the adaptation period.

In conclusion, the shortening of the adaptation period from 14 to 9 or 6 days using only VM as the sole feed additive is not recommended since it compromises carcass fat deposition, disrupts feeding behavior pattern, and does not promote the greater development of both the rumen and cecum epithelium.

## Data Availability Statement

The raw data supporting the conclusions of this article will be made available by the authors, without undue reservation.

## Ethics Statement

The animal study was reviewed and approved by São Paulo State University Ethical Committee for Animal Research (protocol number 154/2016).

## Author Contributions

AR and DM designed the experiment. AR, MS, AS, DE, LF, ED, BD, AN, EC, PS, and CS conducted the experiment. AR, AP, DE, LF, ED, BD, AN, VC, and EC performed the laboratory and data analyses. AR, MA, and DM provided intellectual input. AR, LF, and DM wrote the manuscript. All the authors edited and approved the manuscript for submission.

## Conflict of Interest

The authors declare that the research was conducted in the absence of any commercial or financial relationships that could be construed as a potential conflict of interest.

## Publisher's Note

All claims expressed in this article are solely those of the authors and do not necessarily represent those of their affiliated organizations, or those of the publisher, the editors and the reviewers. Any product that may be evaluated in this article, or claim that may be made by its manufacturer, is not guaranteed or endorsed by the publisher.
